# Itching for Answers: A Comprehensive Review of Cholestatic Pruritus Treatments

**DOI:** 10.3390/biom14101227

**Published:** 2024-09-28

**Authors:** Filippo Gabrielli, Eleonora Crepaldi, Alessia Cavicchioli, Marco Rivi, Arianna Carmen Costanzo, Carmela Cursaro, Pietro Andreone

**Affiliations:** 1 Department of Medical and Surgical Sciences for Children & Adults, University of Modena and Reggio Emilia, 41126 Modena, Italy; 2 Internal and Metabolic Medicine, AOU of Modena-Baggiovara, 41126 Modena, Italy; 3 Postgraduate School of Allergology and Clinical Immunology, University of Modena and Reggio Emilia, 41126 Modena, Italy; 4 Department of Hepato-bilio-pancreatic Surgery and Liver Transplantation, Hautepierre Hospital, Avenue Molière, 67200 Strasbourg, France

**Keywords:** cholestasis, pruritus, drug pipeline, IBAT, fibrates, PPAR agonist, elafibranor, K opioid receptor agonist, cannabinoid

## Abstract

Cholestasis is a clinical and laboratory syndrome indicating impaired bile production or excretion. One of the hallmark symptoms of cholestasis is pruritus. Itch can be severe and debilitating for patients, impacting their quality of life similarly to pain, and, in some cases, it can be refractory. Current therapies like anion exchange resins and rifampicin, offer partial relief but with side effects. Effective, well-tolerated treatments are urgently needed. This literature review examines existing options (bile acid sequestrants, antihistamines, opioid antagonists, sertraline, and rifampicin) and explores novel therapies (monoclonal antibodies, PPAR agonists, and bile-acid-based therapies). We analyze mechanisms, limitations, and adverse effects to aid clinicians and researchers. Novel approaches include monoclonal antibodies to inhibit bile recirculation and PPAR agonists targeting pruritus signaling. Despite the limited current options, ongoing research promises better treatments for cholestatic pruritus, addressing its distressing impact. In summary, cholestasis-associated pruritus poses a significant challenge with limited treatments. Advancements in understanding its pathophysiology offer hope for more effective therapies in the future.

## 1. Introduction

Cholestasis is a pathological condition characterized by a slowdown or interruption in the flow of bile, a fluid produced by hepatic cells. Bile is a fundamental product both for the absorption of metabolites at the intestinal level and for the elimination of substances and toxins; it is involved in the absorption of fat-soluble vitamins, the excretion of lipids, and the elimination of drugs. Although most stimuli that can lead to an alteration in the physiological dynamics of bile excretion are unknown, xenobiotics, exotoxins, endotoxins, and infectious agents can promote the cholestatic state. Additionally, an initial subdivision of cholestatic diseases into intrahepatic and extrahepatic can be considered based on the origin of the obstruction [1]. Pruritus is a symptom that, in some cases, can be very disabling [2]. Chronic pruritus, defined as itching lasting for more than six weeks, can be divided into four groups: of dermatological origin, of systemic origin (cholestasis, leukemia, or chronic renal failure), of neuropathic origin, and of psychogenic origin [3]. Chronic pruritus is one of the most frequent symptoms of primary biliary cholangitis (PBC), the most common cholestatic liver disease, manifesting in over 50% of patients affected by this pathology and limiting their quality of life both physically and psychologically [2,4]. Furthermore, the quality of life of patients with chronic pruritus is similar to that of patients with chronic pain [5]. Although the pathophysiological mechanism of cholestatic pruritus is not yet fully understood, recent research has shed light on various pathways involved, and several drugs have therefore been tested. This study provides a literature review of currently available treatments and drugs under investigation for cholestatic pruritus.

Following the EASL and AASLD Clinical Practice Guidelines (CPGs) from 2009, 2017, 2019, and 2023 focused on the management of cholestatic liver diseases, primary sclerosing cholangitis, and primary biliary cholangitis [3,6,7,8,9], the treatment of cholestatic pruritus is usually managed by combining general measures, such as skin-care strategies, with medication. Skin-care strategies may include using cooler shower water or showering in the morning to avoid night exacerbation and spare some sleeping hours, and using less aggressive soaps and laundry detergents with a complete clothes rinse to eliminate detergent residue after the wash. The use of moisturizers can help prevent dry skin associated with pruritus. The use of creams based on lecithins (phosphatidylcholines) may be beneficial as the phosphatidylcholines contained therein can bind to and neutralize the irritating effects of bile acids when they penetrate the skin. These are empirical recommendations as solid studies evaluating their efficacy are lacking [10]. Psychological intervention and searching for other allergens are suggested. Then, a pharmacological multistep approach is suggested if general measures are insufficient.

## 2. Materials and Methods

We conducted a non-systematic review, following PRISMA guidelines [11], with the following electronic sources: PubMed, Scopus, and ClinicalTrial.gov.

We used the following search words: (“cholestatic pruritus”) OR (“cholestatic itch”) AND (“treatment”). We included free full texts, full texts, classical articles, clinical studies, clinical trials, clinical trial protocols, phase 1 to 5 clinical trials, comparative studies, controlled clinical trials, meta-analyses, multicenter studies, observational studies, practice guidelines, randomized controlled trials, reviews, and systematic reviews published from 2000 to June 2024. Human and in vitro studies were included, and we highlighted data on mortality, survival, and pruritus improvement. We excluded articles not in the English language, studies that did not consider pruritus as an endpoint, and preprints.

The search was conducted as follows: Dr. Gabrielli (F.G.) and Dr. Costanzo (A.C.C.) identified relevant studies by reading the abstracts and searching for additional studies through the reference lists of the selected papers. Then, Dr. Gabrielli (F.G.) and Dr. Costanzo (A.C.C.) independently reviewed the studies by checking titles and abstracts of the articles and decided whether to include each article or not. Non-original articles and off-topic articles were excluded.

### Article Screening and Selection

In the first step, two reviewers (F.G. and A.C.C.) independently evaluated the eligibility of all of titles and abstracts. A study was included in the full-text screening if either reviewer identified the study as potentially eligible or if the abstract and title did not include sufficient information for exclusion. Studies were eligible for full-text screening if they included the data on treatment, dosage, physical and biochemical responses, and the presence of a control group where possible. According to the previously defined inclusion and exclusion criteria, in the second step, the same reviewers independently performed a full-text screening to select articles for qualitative synthesis. Disagreements were resolved via consensus (F.G, A.C.C.) or arbitration (P.A.).

**Figure 1:**
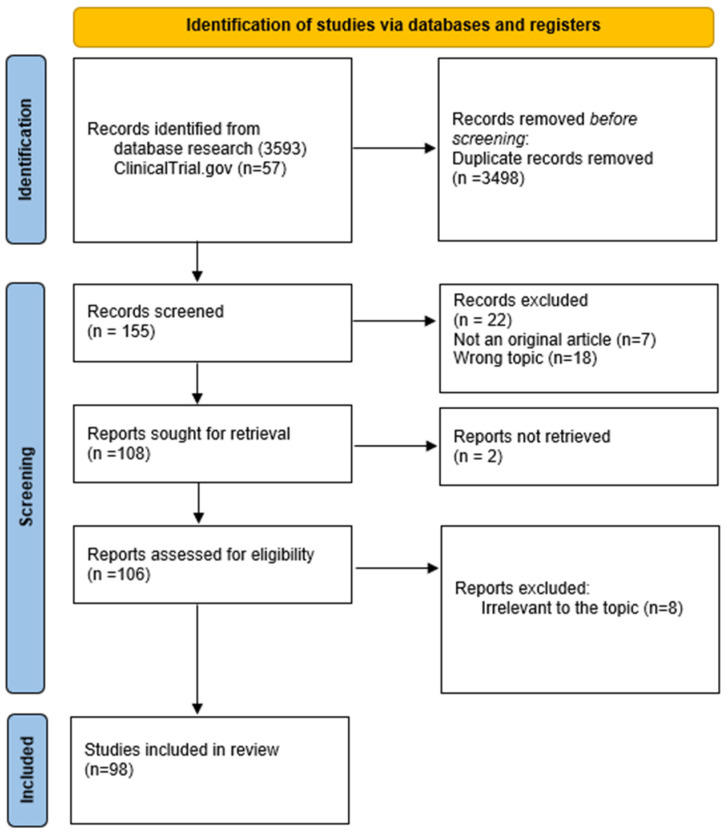
PRISMA flow diagram for studies selection.

A total of 3593 articles were found from PubMed and from Scopus and 57 studies from ClinicalTrial.gov. The flow diagram regarding the selection of articles is reported below (see Figure 1).

## 3. Mechanisms and Key Players in Cholestatic Pruritus

In this section, we will analyze the known pathogenetic mechanisms underlying the development of cholestatic pruritus.

### 3.1. Cholestasis

**Figure 2:**
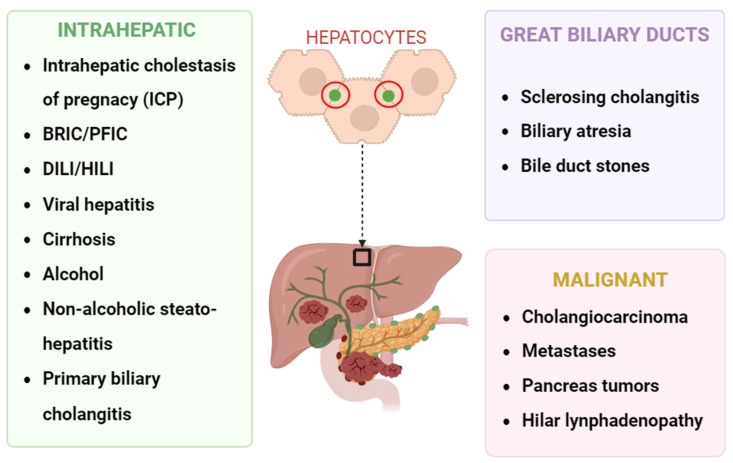
Most common causes of cholestatic pruritus. BRIC = benign recurrent intrahepatic cholestasis, PFIC = progressive familial intrahepatic cholestasis, DILI = drug-induced liver injury, HILI = herb-induced liver injury. Primary biliary cholangitis (PBC) and primary sclerosing cholangitis (PSC) can present without abnormalities on cholangiography.

Due to the complex range of functions that bile flow affects, its impairment can affect various aspects of digestion, body function, and detoxification [12]. The first signs of cholestasis often appear as alterations in blood tests, with an increase in plasma levels of total bile acids, total bilirubin, alanine aminotransferase (ALT), alkaline phosphatase (ALP), gamma glutamyl transpeptidase (GGT), and aspartate aminotransferase (AST). The principal symptoms of cholestasis are jaundice (caused by the deposition of bilirubin in the skin), dark urine (caused by the kidney’s excretion of bilirubin), acholic stools (caused by reduced bilirubin in stools), steatorrhea (caused by the excretion of fat in feces), fatigue (a sense of exhaustion that heavily affects the quality of life), and pruritus [13]. A list of cholestatic diseases is presented in Figure 2.

### 3.2. Cholestatic Pruritus

Pruritus is a symptom and is frequently the first manifestation of some cholestatic diseases. The condition has a wide range of intensity from a small disturbance to a disabling disease, with loss of sleep, depression, and compromised quality of life [2,4]. Cholestatic pruritus differs from other types of itching in terms of onset, duration, localization, and response to therapy, especially to antihistamine therapy. It is primarily present in pathologies with lesions of small bile ducts rather than those with obstruction of large bile ducts [14]. For example, in primary sclerosing cholangitis, one of the cholestatic liver diseases, itching is present in 30% to 50% of patients [15,16]. A study conducted by Kim N. van Munster et al. on 137 PSC patients showed how itching in this type of patient fluctuates both during the day and in different seasons, with peaks of itching in the evening and in winter [17]. This last finding was also reported by Oeda et al. in patients with cholestatic liver disease [18]. The locations of cholestatic itching are various; typically, patients report itching on the back, calves, abdomen, hands, and soles of the feet, although generalized itching is also possible [18,19]. Cholestatic pruritus often occurs in the absence of jaundice.

This issue can arise from intrahepatic cholestasis due to damage to hepatocyte secretion (such as progressive familial intrahepatic cholestasis (PFIC), benign recurrent intrahepatic cholestasis (BRIC), and intrahepatic cholestasis of pregnancy (ICP)) or damage to intrahepatic bile ducts (such as primary and secondary sclerosing cholangitis (PSC/SSC), primary biliary cholangitis (PBC), and pediatric cholestatic syndromes) [18]. While less intense and less frequent, cholestatic pruritus can also be present in extrahepatic cholestasis due to obstruction resulting from a compromise in the biliary tree (such as a tumor or lymph node compression) or intraductal obstruction (such as PSC/SSC, choledocholithiasis, cholangiocellular carcinomas, or biliary atresia).

### 3.3. Mechanism of Pruritus Development in Cholestasis

The mechanism of pruritus development in cholestatic liver disease was extensively studied but is still poorly understood. A discussion of the pathophysiology of pruritus is beyond the scope of this review; however, recent articles have provided a discursive account of it [14,20]. We will examine some pathways involved in the transmission of pruritus to elucidate the therapeutic targets that will be discussed later.

Although itching has long been considered the “little brother of pain” because it was thought that the transmission of itching occurred through the same nerve fibers as pain, this concept changed in the 1990s. In fact, a subgroup of non-myelinated meccano-insensitive C-nociceptor nerve fibers sensitive to itching was discovered, with their endings located in the skin and cerebrum areas such as the supplementary motor cortex, primary sensory cortex, parietal lobe, and cingulate gyrus [3,21]. However, itch and pain share some characteristics, including the activity of the transient receptor potential cation channel subfamily V member 1 (TrpV1), also known as the capsaicin receptor and the vanilloid receptor 1 [22]. This receptor can be activated by lysophosphatidic acid (LPA) [23]. Another interesting relationship between itch and pain is that the latter inhibits the former at the level of the spinal cord; this is due to the presence of inhibitory interneurons in the dorsal horn of the spinal cord. Other neurotransmitters and receptors have been identified as possible mediators of itch transmission, including gastrin-releasing peptide (GRP), natriuretic polypeptide b (NPPB), murine Mas-related G-protein-coupled receptor member X4 (MRGPRX4), Takeda G-protein-coupled receptor (TGR5), and farnesoid X receptor (FXR). NPPB appears to act between the primary and secondary neurons by inducing the release of GPR from the secondary neuron via stimulation [14]. Knockout mice for the NPPB gene, which encodes natriuretic polypeptide b, are less sensitive to pruritogenic stimuli [24]. The role of TGR5, on the other hand, is debated as it may act at the level of spinal transmission of itch, and experiments on mice with overexpression of this receptor show intense itch after intradermal administration of unconjugated bile acids compared to mice knockout for this receptor. However, the injection of a non-bile acid agonist of TGR5 did not induce itching, and the doses of bile acids administered were extremely high [25,26]. In vitro experiments have demonstrated the activation of MRGPRX4 expressed by human sensory neurons, while in vivo experiments in mice have shown contrasting results [27,28,29]. A recent finding is related to the agonists of FXR, such as obeticholic acid used in some trials for non-alcoholic steatohepatitis (NASH) and primary biliary cholangitis (PBC), where itching was reported in a third of patients. However, the mechanism leading to the genesis of itching in this case has not yet been demonstrated [30,31,32]. It has been hypothesized that the farnesoid X receptor (FXR) may induce the bile salt export pump (BSEP), a transporter capable of moving bile acids from hepatocytes to the bile canaliculi, while simultaneously reducing the activity of the sodium taurocholate cotransporting polypeptide (NTCP), thereby maintaining low levels of bile acids within hepatocytes [33]. Additionally, during cholestasis, FXR induces multidrug-resistance-associated protein 4 (MRP4) as an adaptive mechanism to promote the efflux of bile acids into the bloodstream [33]. This could explain the increased pruritus observed with the use of FXR agonists. Non-steroidal agonists of FXR, such as tropifexor and cilofexor, have also shown potential pruritogenic effects [34,35]. Although the molecular mechanisms underlying pruritus in cholestasis are still being investigated, it is evident that treatments leading to a reduction in serum bile acids, such as nasobiliary drainage, apical sodium-dependent bile acid transporter inhibitors, and plasmapheresis, result in a decrease in pruritus [36].

### 3.4. Pruritogens

The knowledge of pruritogens, substances capable of inducing itch, is of fundamental importance for understanding pruritic pathologies and for the development of new therapies. Several substances have been identified as suspected pruritogens in cholestatic diseases. Among these, we can list bile acids, bilirubin, endogenous opioids, histamine, serotonin, progesterone, estrogen, and LPA.

#### 3.4.1. Bile Acids

Bile acids are the end product of cholesterol catabolism, which are subsequently transformed by intestinal microbiota. Bile acids can activate specific extracellular and intracellular receptors that are not activated by conventional steroids, including TGR5, muscarinic receptors M2 and M3, FXR, and MRGPRX4 [37]. Although it has been believed for millennia that cholestatic pruritus is due to bile acids themselves, there are some observations that suggest their secondary role. In fact, it has been noted that pruritus is a manifestation of cholestatic diseases, despite bile acid levels generally being only slightly above normal upper limits, and that in some diseases, such as sodium taurocholate cotransporting polypeptide (NTCP) deficiency, an inborn error of bile acid metabolism resulting in bile acid levels more than 80 times the normal, there is no pruritus [38,39,40,41,42]. However, the role of bile acids in the mechanism of pruritus should not be understated, especially given the limited number of studies that have specifically examined their individual components and forms in plasma. The impact on itching could be dependent on these factors, with unconjugated, non-sulfated, and non-glucuronidated hydrophobic bile acids exerting a significantly stronger irritative effect on nerve endings.

#### 3.4.2. Bilirubin

Bilirubin is a product of the catabolism of hemoglobin. Unconjugated bilirubin is hydrophobic and therefore transported in the blood associated with albumin; in the liver, glucuronidation occurs, making it soluble and ready to be secreted in bile. At the intestinal level, it is transformed into urobilinogen, which can be eliminated with feces and reabsorbed. In vitro experiments have shown how bilirubin can activate MRGPRX4, and knockout mice for the gene that encodes this receptor show less itching [43]. However, genetic diseases such as Crigler–Najjar syndrome type 1 and Dubin–Johnson syndrome, which involve hyperbilirubinemia, do not manifest significant itching. As for bile salts, we may not yet know all the pruritogenic pathways that involve bilirubin.

#### 3.4.3. Steroids

Several studies have investigated a possible pruritogenic action of steroids (e.g., progesterone and estrogen) particularly in patients with intrahepatic cholestasis of pregnancy (ICP). It has been observed that there is an increase in certain pregnanediol sulfates and that 5β-pregnanediol-3α,20α-diol sulfate (PM3S) can activate TGR5, inducing itching in both mice and humans, while TGR5 knockout mice do not show itching [25,44].

#### 3.4.4. LPA and Autotaxin

LPA is a neuronal activator and is one of the major suspects in the genesis of cholestatic pruritus as it is capable of activating TRPV1 channels of C-fiber endings, leading to pruritus [23]. The genesis of LPA involves the enzyme autotaxin (ATX), which generates LPA using phosphatidylcholine (LPC) as a precursor. Elevated levels of ATX have been found in patients with cholestasis and pruritus, while patients with cholestasis alone still had higher values than healthy controls [45]. In addition, it has been noted that the serum levels of ATX were in line with the reaction to various remedial measures, such as the anion exchange resin, colesevelam; the potent activator of pregnane X receptor (PXR), rifampicin; nasobiliary drainage; and MARS treatment [46]. Nevertheless, elevated levels of LPA may also be present in physiological pregnancies and neoplasms [45]. Recent studies have shown that LPC can activate TRPV4, that this leads to pruritus, and that the use of an autotaxin inhibitor halved scratching in mice subsequently injected with LPC [47]. Finally, it has been observed that various bile compounds that accumulate during cholestasis, such as 3-OH sulfated bile salts and sulfated progesterone metabolites, inhibit ATX activity, but this results in increased ATX expression through a feedback regulation mechanism [48]. Further studies on the role of ATX and LPA need to be conducted.

#### 3.4.5. Endogenous Opioids

The endogenous opioid system may play a minor role in the genesis of cholestatic pruritus. One of the first considerations is that the liver plays an important role in the metabolism of opioids, leading to their accumulation and excretion, and that in patients with liver damage, plasma levels of opioids are increased. Several studies have demonstrated the role of opioids in the genesis of pruritus. In particular, it has been noted that the initiation of therapy with opioid antagonists can lead to withdrawal syndrome in patients with cholestatic pruritus and that mu-opioid receptor antagonists moderately reduce cholestatic pruritus [49,50,51,52,53]. In addition, spinal opioid administration induces pruritus [54], and the expression of mRNA precursors of endogenous opioids has been found in the livers of mice in which cholestasis was induced [55]. Therefore, it is believed that mu receptor agonists induce a pruritogenic reaction, while kappa receptor agonists reduce pruritic activity [56]. However, no correlation has been found between the intensity of pruritus and endogenous opioid levels or between the mu-opioid receptor/kappa-opioid receptor ratio and cholestatic pruritus [56].

#### 3.4.6. Serotonin

The mechanism by which serotonin induces pruritus is related to the activation of 5-hydroxytryptamine (HT) receptors. It is known that intradermal administration of serotonin induces pruritus and that selective serotonin reuptake inhibitors lead to an improvement in the perception of cholestatic pruritus [56,57]. However, an increase in pruritus has not been found in cases of elevated levels of serotonin, and the 5-HT type 3 inhibitor ondansetron did not show a statistically significant reduction in pruritus [53,58,59,60].

## 4. Recommended Therapies for Cholestatic Pruritus

**Figure 3:**
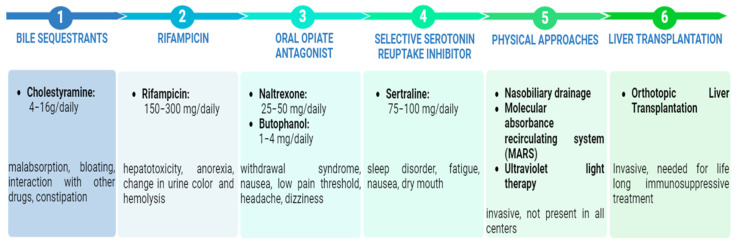
Multistep approach for cholestatic itch treatment and adverse events.

The guidelines recommend a multistep approach to the pharmacological treatment of pruritus. In the following section, we will discuss the drugs suggested by the different guidelines based on their pharmacological category. Figure 3 shows all treatments proposed by EASL and AASLD for cholestatic pruritus [3,6,8,9]. The first step generally involves the use of bile acid sequestrants, followed by the use of rifampicin, opioid antagonists, and serotonin reuptake inhibitors up to the removal of catabolites through the use of physical approaches and liver transplantation. However, the latest EASL guidelines on genetic cholestasis liver disease propose a different sequence from that reported in previous guidelines. Specifically, they recommend the successive use of fibrates, ursodeoxycholic acid, rifampicin, and ileal bile acid transport inhibitors (IBATs). Subsequently, as less commonly used drugs, they suggest cholestyramine, sertraline, naltrexone, and chaperones [61]. The following discussion will focus on the classes of drugs, their mechanism of action, and the studies conducted on the therapies recommended by the guidelines.

### 4.1. Bile Sequestrants

Cholestyramine, colestipol, and colesevelam are anion exchange resins widely used for the treatment of pruritus in PBC and are listed in both European and American guidelines as first-line treatments for cholestatic pruritus [3,7,9]. The mechanism of action involves the binding of bile acids preventing the reuptake in the terminal ileum, thus removing the pruritogens by stopping the enterohepatic circulation. This effect induces the synthesis of cholecystokinin that can antagonize the opioid receptors, leading to relief [62]. Cholestyramine is a first-line treatment in PBC and a third-line treatment in ICP [14].

Cholestyramine should be administered in a dose of 4 g up to four times daily, at least 20 min before meals and 4 h before other medications [3,63]. To help minimize side effects (i.e., nausea, bloating, and/or constipation), it is recommended to start the therapy at lower doses and progressively increase the dose if needed during a period of weeks to months. Cholestyramine may exhibit poor compliance due to its taste, which can be masked by mixing it with an oral vehicle.

Its beneficial effect is reported in small uncontrolled case series where pruritus improved within 4 to 14 days [64,65]. The clinical experience with cholestyramine is typically good: to date, there are no studies confirming the effectiveness of cholestyramine in pruritus.

Colestipol is another bile acid sequestrant approved for the treatment of LDL hyperlipemia that is used off-label as a therapy for cholestatic pruritus and could be an alternative, but in this case, the clinical experience is limited.

Colesevelam is another bile sequestrant with a 7-fold higher bile acid-binding capacity. In a randomized controlled trial on 35 patients, it did not demonstrate clinical efficacy in reducing pruritus despite a significant reduction in serum bilirubin levels, and its efficacy remains uncertain [66]. A summary of the bile sequestrants used for cholestatic pruritus is provided in Table 1.

### 4.2. Pregnane X Receptor (PXR) Agonists

Following the EASL 2017 guidelines, the second-line treatment is rifampicin [3]. Rifampicin is also recommended during the third trimester in women with ICP, as it has been proven to be effective and safe, and in general, it is safe throughout pregnancy [6,67]. A multicenter, randomized, open-label, controlled study is currently ongoing to evaluate whether rifampicin is superior to UDCA in reducing pruritus in ICP [68]. The EASL and AASLD guidelines for sclerosing cholangitis strongly recommend the use of rifampicin in cases of moderate or severe pruritus [6,8]. The exact mechanism by which rifampicin reduces pruritus is not yet fully understood, but it has been suspected that the activation of PXR may induce a reduction in ATX transcription [46]. The efficacy of rifampicin in cholestatic pruritus has been demonstrated in four RCTs [69,70,71,72] and confirmed by two meta-analyses [73,74]. A recent RCT comparing the use of sertraline versus rifampicin did not show significant differences in the improvement of pruritus between the two groups [75]. Caution should be exercised as cases of hepatitis related to the use of rifampicin have been reported, especially after prolonged treatment, and therefore monitoring of liver function is recommended in patients taking rifampicin [76]. The recommended dose is 10 mg/kg daily, starting from 150 mg orally daily or twice daily titered up to 600 mg daily. The escalation must be guided by the clinical need and the absence of hepatoxicity. The expected time of effect is 2 days in the average patients [9]. Less common adverse events, including hemolytic anemia, leukopenia and agranulocytosis, thrombocytopenia, renal impairment, severe dermatologic reactions, and anaphylaxis, have been reported [77]. A summary rifampicin in cholestatic pruritus is provided in Table 2.

### 4.3. Opioid Antagonists

Oral opiate antagonists are the third-line treatment [3]. The EASL guidelines specifically recommend the use of naltrexone. Among the most studied and utilized members in this class are naltrexone, naloxone, nalfurafine, and nalmefene; generally, the sedative activity for itch is effective. As previously explained, the mechanism of action occurs at the level of inhibitory interneurons in the spinal cord.

Naltrexone has been evaluated in patients with cholestatic pruritus compared to a placebo in two RCTs, demonstrating effectiveness in reducing pruritus and good tolerance [78,79]; however, its use is off-label. Naloxone is only available in an intravenous formulation and is recommended for selected hospital settings. A double-blind, placebo-controlled RCT conducted on 29 patients with cholestatic pruritus demonstrated a statistically significant reduction in pruritus in the group receiving naloxone [50,80].

Nalmefene is an oral opioid antagonist, the safety and tolerability of which have been confirmed through an RCT study demonstrating low toxicity, good bioavailability despite oral administration, greater potency than naloxone, and longer half-life [51]. However, several meta-analyses have shown that the potency of opioid antagonists in reducing cholestatic pruritus is inferior to rifampicin [73].

Nalfurafine is a kappa opioid receptor agonist and has been evaluated for uremic pruritus in 337 patients undergoing hemodialysis, demonstrating a reduction in pruritus in patients refractory to other therapies [81]. Regarding cholestatic pruritus, a double-blind RCT was conducted with 318 subjects, resulting in an improvement in the perception of pruritus with a concomitant reduction in the Visual Analog Scale (VAS) [82]. Two other studies have evaluated the effectiveness of nalfurafine. An observational study on 24 patients with chronic liver disease reported a statistically significant reduction in VAS, with 71% of patients reporting an improvement in pruritus [83], and a prospective single-arm study on 44 patients with PBC demonstrated a statistically significant reduction in pruritus scores [84].

Another kappa opioid receptor agonist, butorphanol, is used in pruritus associated with atopic dermatitis, but RCTs have not yet been conducted in cholestatic pruritus. However, a case series of eight patients reported a 62.5% benefit rate from the use of the drug [85].

The main side effects of naltrexone therapy include dizziness, headache, nausea, vomiting, and rare cases of hepatotoxicity. The most important side effect is the withdrawal syndrome, which is caused by the displacement of opioids from µ and κ opioid receptors. The typical duration of this side effect is around 2 or 3 days in the majority of cases, and great attention must be paid to patients who have chronic pain as they may experience pain flares after starting the therapy [77,78,86,87]. Table 3 presents the most common opioid antagonists used for cholestatic itch.

### 4.4. Modulators of Serotoninergic Pathways

The following drugs belong to this category: sertraline, ondansetron, phenobarbital, propofol, and gabapentin; however, only sertraline is a fourth-line drug indicated by the EASL guidelines [3].

Serotonin acts as an antidepressant through selective serotonin reuptake inhibition. The antipruritic effect is likely mediated by an alteration in the concentration of neurotransmitters in the central nervous system (CNS) [77]. The antipruritic effect seems to be independent of the antidepressant effect [88]. Sertraline has been evaluated in three trials [75,88,89]. In the first study, 21 patients with pruritus associated with liver disease were randomized to receive a placebo or sertraline as a first-line treatment. Sertraline was found to be effective and well-tolerated in the treatment of pruritus at a dose of 75–100 mg/day [88]. The second study was conducted on 20 patients with pediatric Alagille syndrome and demonstrated a statistically significant reduction in pruritus [89]. The third trial, a single-blinded randomized controlled trial that enrolled 36 patients with primary sclerosing cholangitis (PSC) and primary biliary cholangitis (PBC), demonstrated a non-inferiority against rifampicin [75]. Sertraline was associated with better liver safety, as evidenced by less elevation of hepatobiliary enzymes compared to rifampicin [75].

Ondansetron selectively antagonizes 5-hydroxytryptamine 3 receptors tested in three RCTs [58,59,60], showing no statistical difference in ameliorating pruritus compared to a placebo.

Phenobarbital and propofol have been used as hypnotics for refractory pruritus, but their use is generally not recommended due to a lack of efficacy and significant side effects as marked sedation and somnolence, which limits its use [9,69,90,91].

The mechanism of action of gabapentin in reducing pruritus may be related to its ability to increase the threshold of nociception. However, an RCT conducted on 16 women with pruritus associated with liver disease failed to demonstrate a reduction in pruritus compared to a placebo [92]. Table 4 summarizes the most common modulators of serotonergic pathways used for cholestatic itch.

### 4.5. Physical Approaches

The aim of physical approaches is to remove the pruritogenic substance. Physical approaches proposed for cholestatic pruritus have been minimally studied, with the available literature limited to small, controlled studies. This is primarily due to logistical challenges, such as the need for highly specialized personnel, appropriate facilities, and specific materials. Consequently, the widespread adoption and validation of these interventions remain constrained, highlighting the necessity for larger, more comprehensive trials to better assess their efficacy and practicality in clinical settings. Nasobiliary drainage, the molecular absorbance recirculating system (MARS), ultraviolet (UV) light therapy, charcoal hemoperfusion, and plasmapheresis can be included among the physical approaches.

The MARS is an artificial extracorporeal liver support (ECLS) capable of potentially removing both water-soluble toxins and those soluble in albumin (such as bilirubin) [93]. The MARS employs an additional circuit compared to standard extracorporeal circuits, which includes a 20% albumin-containing dialysis medium [94]. Different studies involving patients with cholestatic liver disease due to PBC, or non-anastomotic strictures, ductopenic graft rejection, and refractory pruritus, were conducted and showed an immediate reduction in pruritus, a benefit that lessened in the following months and often led to the reuse of the method [95,96,97,98]. Success in improving refractory cholestatic pruritus with the MARS has also been demonstrated in the pediatric population [99,100]. The only significant side effect was a temporary reduction in platelet count and hemoglobin level after treatment, but the hematic levels returned to baseline within a month. The principal limitations of this treatment are the high cost and the availability and expertise of clinical personnel.

UV light therapy is used in various pruritic conditions, such as atopic dermatitis. In neonates with jaundice, it is capable of increasing urinary excretion by increasing the hydrophilicity of bilirubin [101]. Some authors have reported an alteration in cytokine release in the skin and blood, as well as histological changes such as a reduction in Langerhans cells, degeneration of Schwann cells, and T-suppressor lymphocytes [102]. UV light therapy has been undertaken in patients with pruritus due to various cholestatic conditions (PBC, PSC, drug-induced liver injury, and post-OLT) refractory to conventional medical therapies reporting a reduction in pruritus [103,104]. However, the studies on phototherapy were conducted on small populations and often are case reports. Further studies to understand the mechanism of action and the efficacy of UV light therapy are required.

The studies on the use of plasmapheresis in cholestatic pruritus are limited. Cohen et al. used plasmapheresis as a remedy for pruritus unresponsive to pharmacological therapies in five patients with PBC, showing an improvement in pruritus but side effects such as transient hypotension and urticaria [105]. Another study on two cases of severe cholestatic pruritus during pregnancy showed the safety and efficacy of the treatment [106]. Attention should be given to the use of plasmapheresis in patients with advanced liver cirrhosis (Child–Pugh C) as plasma removal can lead to a biosynthetic overload on hepatocytes.

Nasobiliary drainage (NBD) involves the placement of a catheter in the common bile duct during endoscopic retrograde cholangiopancreatography (ERCP). The rationale for this procedure is to prevent the enterohepatic recirculation of bile and bile acids, thereby avoiding the reabsorption of 90% of these substances. The largest study evaluating this procedure was a multicenter retrospective study that included 27 patients with cholestatic pruritus treated with NBD, with a median age of 41 years [104,107]. A statistically significant difference in pruritus perception was observed between before and after the procedure. However, there were nine cases of post-procedural pancreatitis and one case of post-ERCP cholangitis reported [107].

Charcoal hemoperfusion involves extracorporeal filtration of the blood. In a retrospective study, charcoal hemoperfusion provided significant but temporary relief from pruritus [108]. Adverse events were prevalent and included dialyzer reactions such as pain, fever, nausea, and hypotension, with 15% of the participants not completing the study due to these reactions [108]. Table 5 summarizes the most common physical approaches used for cholestatic itch

### 4.6. Liver Transplantation

In general, orthotopic liver transplantation (OLT) is considered the final option for end-stage liver disease and acute liver failure. However, it is suggested as a last resort for intractable cholestatic pruritus that has not responded to other treatments [3,6,8]. The decision to undertake this major surgical intervention typically follows a careful evaluation of patient’s condition and prognosis, considering the quality of life, the extent of liver damage, and the severity and refractoriness of pruritus. Liver transplantation can be curative for cholestatic conditions, with reports indicating complete resolution of pruritus post-operatively in a vast majority of cases. This dramatic relief from pruritus is likely due to the elimination of the underlying cholestatic condition and the restoration of normal bile flow, hence effectively removing the pathophysiological triggers of pruritus. However, recurrence of the original disease in the transplanted liver can occur, especially concerning autoimmune hepatitis, PBC, and PSC [109]. In a comparative study of 157 patients with end-stage PBC or PSC who underwent liver transplantation, itching was significantly reduced after liver transplantation [110].

## 5. Drug Pipeline

In recent years, the development of new molecules for the therapy of pruritus in cholestatic diseases has grown due to the gradual improvement in knowledge of the mechanisms of pruritus. Below, we will report the molecules that are in pharmacological trials for cholestatic pruritus.

### 5.1. Peroxisome Proliferator-Activated Receptor (PPAR) Agonists

Peroxisome proliferator-activated receptors (PPARs) are intracellular transcription factors that are involved in numerous pharmacological and physiological processes, including gene expression, inflammation, carcinogenesis, and metabolic pathways [111]. PPARs form a heterodimeric complex with the retinoid X receptor and interact with particular DNA sequences to govern objective genes once they attach to their ligands [76]. PPARα has various functions, including regulating the metabolism of bile acids in different ways: inhibiting bile acid synthesis or increasing secretion, reducing bile toxicity, and detoxifying bile acids [112,113,114,115,116,117]. Fibrates act as agonists of PPAR α, γ, and δ and are currently used in the treatment of hyperlipidemia [118]. Fibrates have been studied for their anti-inflammatory and protective effects on the bile ducts in both PBC and PSC, but for cholestatic pruritus, the studies are limited. In 2021, the FITCH (Fibrates for Itch) trial, a double-blind, placebo-controlled RCT, evaluated the efficacy of bezafibrate in modulating moderate-to-severe pruritus in patients with PSC, PBC, and secondary sclerosing cholangitis (SSC) and showed a reduction of more than 50% in pruritus [119]. However, it is important to note that the treatment period was only 21 days, that patients with Child–Pugh C were not included, and that patients with renal insufficiency were excluded. The BEZURSO trial, a placebo-controlled RCT in which 100 PBC patients who were unresponsive to UDCA therapy were randomly assigned to receive 100 mg of bezafibrate/daily or a placebo, showed as a secondary outcome a difference in change from the baseline in the 24-month itch intensity score between bezafibrate and a placebo of −95% [CI95% −241–50%] [120]. Two recent meta-analyses have confirmed the reduction in pruritus in patients who received bezafibrate [121,122]. The most frequently reported side effects were myalgia, reduced glomerular function, and elevated aminotransferase levels. The latest EASL guidelines on PSC recommend bezafibrate as a first-line therapy for pruritus [6]. Although the AASLD guidelines for PSC also describe an improvement in pruritus, the drug has not yet been approved for this purpose by the Food and Drug Administration for use in the USA. Currently, several trials are underway to investigate the use of bezafibrate in cholestatic diseases, which are reported in the Table 6. Fibrates have been extensively studied in non-RCT studies with prospective cohorts, but few studies have evaluated their efficacy for pruritus. A recent meta-analysis indicated that fibrates are capable of mitigating cholestatic pruritus [123].

Seladelpar is a potent PPAR δ agonist, the anticholestatic activity of which has been tested in several randomized controlled trials (RCTs). Its anticholestatic action is caused by the inhibition of several mechanisms, including the synthesis and absorption of cholesterol through the reduction in Niemann–Pick C1-like protein, as well as the reduction in bile acid synthesis via a decrease in CYP7A1 expression and a reduction in 7α-hydroxycholesterol and 7α-hydroxy-4-cholesten-3-one [124,125]. As for pruritus, a reduction in itch has been reported with a decrease in VAS, with a percentage of patients reporting an improvement in itching ranging from 58% to 93% depending on the drug dose [126]. Other studies on seladelpar in the treatment of PBC have evaluated changes in pruritus as a secondary outcome compared to baseline; among those that provided results, a reduction in pruritus is often reported, although a recent meta-analysis concluded that there is evidence to suggest that the reduction in pruritus may be due to the experimental drug [NCT03602560], [122,124]. Seladelpar has been approved by the FDA for the treatment of primary biliary cholangitis (PBC) in combination with ursodeoxycholic acid (UDCA) in adults who have demonstrated an inadequate response to UDCA or as a monotherapy in patients who are intolerant to UDCA. However, it is currently not indicated for the treatment of cholestatic pruritus, and there are no data supporting its use for improving survival or preventing liver decompensation.

Elafibranor is a dual PPAR α/δ agonist that was initially used in non-alcoholic steatohepatitis, demonstrating a reduction in steatosis, inflammation, and hepatic fibrosis [127]. Regarding cholestatic itching, it was evaluated in the GENFIT study for 12 weeks in patients with PBC and an incomplete response to UDCA [128]. In this study, a reduction in itching was reported in the subgroups of patients with pruritus who were taking elafibranor, as measured using the VAS scale. However, the sample size was limited to 10 patients per group. No worsening of itching related to the administration of elafibranor was observed [128]. The ELATIVE study evaluated the reduction in pruritus as a secondary outcome in patients with primary biliary cholangitis (PBC) who were either non-responsive or intolerant to UDCA [129]. The results were mixed. Patients with moderate to severe pruritus receiving elafibranor showed a statistically significant reduction in itching, as measured with the pruritus domain of the PBC-40 Quality of Life (QOL) questionnaire (least-squares mean difference: −2.3; 95% CI: −4.0 to −0.7) and the 5D itch scale (least-squares mean difference: −3.0; 95% CI: −5.5 to −0.5) [129]. However, when using the Worst Itch Numerical Rating Scale (NRS), no significant differences in pruritus reduction were observed between the elafibranor group and the placebo group after 52 weeks (−1.93 vs. −1.15; difference, −0.78; 95% CI, −1.99 to 0.42; *p* = 0.20) [129]. Two pharmacological trials evaluating the efficacy of elafibranor in reducing cholestatic itching are currently ongoing; they are reported in Table 6. In June 2024, elafibranor received accelerated approval from the FDA for use in patients with primary biliary cholangitis (PBC) who are intolerant to ursodeoxycholic acid (UDCA) or have an inadequate response to it. Similar to seladelpar, there is currently no indication for pruritus associated with cholestasis.

**Table 6:** Main clinical studies on fibrates in cholestatic itch.

Drug	Reference NCT Number	Study Type (ST), Population (P), and Dosage (D)	Results	Adverse Effects (AEs)	Limitations/Comments
**BEZAFIBRATE**	de Vries E. et al. (FITCH) [119]	ST: double-blind, placebo-controlled RCT P: 84 with PBC or primary/secondary sclerosing cholangitis and VAS ≥ 5 D: bezafibrate 400 mg/daily vs. placebo	More than 50% reduction in pruritus in 55% of patients treated with bezafibrate compared with a pruritus reduction in 13% of patients from the placebo group	Myalgia, reduced renal function, and elevation of aminotransferases	Duration of the study was 21 days
	Corpechot C. et al. [120]	ST: double-blind, placebo-controlled RCT P: 100 patients with PBC and inadequate response to UDCA D: bezafibrate 400 mg/daily vs. placebo for 24 months	Difference in change from the baseline in the 24-month itch intensity score between bezafibrate and placebo of −95% [95% CI −241% to −50%]	Myalgia, reduced renal function, and elevation of aminotransferases	Small sample size, no histological data
	Efficacy and Safety of Bezafibrate 400 mg and Bezafibrate 200 mg as Adjunctive Treatments in Patients With Primary Biliary Cholangitis and Non-optimal Biochemical Response to Ursodeoxycholic Acid Therapy (BEZURSO 2) NCT06443606	ST: double-blind, placebo-controlled RCT P: estimated 108 patients with PBC and a non-optimal biochemical response to UDCA D: bezafibrate 400 mg/daily and placebo of bezafibrate 200 mg vs. bezafibrate 200 mg and placebo of bezafibrate 400 mg vs. placebo of bezafibrate 400 mg and placebo of bezafibrate 200 mg for 96 weeks	Not yet recruiting	Not available yet	Not available yet
	Efficacy of 24-Month of Bezafibrate in Primary Sclerosing Cholangitis With Persistent Cholestasis Despite Ursodeoxycholic Acid Therapy (BEZASCLER) **NCT04309773**	ST: double-blind, placebo-controlled RCT P: 104 with severe PSC and persistent cholestasis despite UDCA **D**: bezafibrate 400 mg/daily + UDCA 15–20 mg/kg/daily vs. placebo + standard UDCA therapy	Ongoing	Not available yet	Not available yet
	Use of Bezafibrate in Patients With Primary Biliary Cirrhosis to Archive Complete Biochemical Response in Non-responders NCT02937012	ST: double-blind, placebo-controlled RCT P: 34 with PBC with suboptimal response to UDCA D: bezafibrate 200 mg twice/daily + UDCA 13–15 mg/kg/die vs. placebo + UDCA 13–15 mg/kg/daily	No result posted	No result posted	Among other outcomes, pruritus evaluation is conducted via the use of visual analogue scales every 3 months for 12 months
	A Study to Assess Efficacy and Safety of Bezafibrate in Patients With Primary Biliary Cholangitis NCT04751188	ST: double-blind, placebo-controlled RCT P: 11 patients with PBC with suboptimal response to UDCA D: bezafibrate 200 mg twice/daily + UDCA 13–15 mg/kg/die vs. placebo + UDCA 13–15 mg/kg/daily	No results posted, still ongoing	No result posted	Among secondary outcomes, pruritus evaluation is conducted via the use of visual analogue scales
**FENOFIBRATE**	Levy C et al. [130]	ST: Open-label pilot study P: 20 patients with PBC with incomplete response to UDCA D: fenofibrate 160 mg/daily for 48 weeks	Of 11 patients that reported pruritus at the baseline, 44% remained stable, 28% improved, and 28% deteriorated during the study	Heartburn, elevated aminotransferase, nausea, arthralgias, weight gain	Pruritus was not an endpoint of the study, and it was recorded at a scale from 0 to 3. Small population
**SELADELPAR**	Kremer AE, et al. [126] NCT02955602	ST: Open-label randomized with a 44-week extension P: 121 patients with PBC with incomplete response to UDCA D: seladelpar 2 mg/daily vs. seladelpar 5 mg/daily vs. seladelpar 10 mg/daily for 52 weeks	A marked decrease in pruritus was observed after 2 weeks of treatment. After 52 weeks, a significant reduction in pruritus was recorded using the VAS in 58% and 93% of patients treated with seladelpar at doses of 5 mg and 10 mg, respectively Additionally, both the PBC-40 score and the total 5D itch score showed statistically significant reductions in the seladelpar group	Upper respiratory tract infection, myalgia, headache, arthralgia; less frequently: pneumonia, febrile neutropenia, angina pectoris, upper abdominal pain, syncope	Not placebo-controlled
	Jones D et al. [124] NCT02609048	ST: double-blind, placebo-controlled, multicenter RCT P: 41 patients with PBC with inadequate response to UDCA D: seladelpar 50 mg/daily vs. seladelpar 200 mg/daily vs. placebo for 18 weeks	Study was stopped early for grade 3 increases in aminotransferase probably due to high doses of seladelpar. Increased pruritus in 16% of patients. Despite that, seladelpar did not appear to be associated with drug-induced or worsened pruritus	Pruritus (16%), nausea (13%), diarrhea (10%) and dyspepsia, muscle spasms, myalgia, dizziness, and hepatitis	High experimental drug dosage, study stopped early, phase 2 study
	Mayo MJ et al. [131] NCT03301506	ST: interventional, non-randomized, open-label P: 104 patients with PBC that completed NCT02955602 or NCT03602560 D: seladelpar 5 mg/daily vs. seladelpar 10 mg/daily for 60 months	Itching was not assessed as a primary outcome	The most common adverse effect was itching, observed in 24.6% of patients	Effects of seladelpar on patient-reported pruritus was a secondary outcome, not placebo-controlled
	Hirschfield MG et al. [132] NCT03602560	ST: double-blind, placebo-controlled, multicenter RCT P: 265 patients with PBC with inadequate response to UDCA D: seladelpar 5–10 mg/daily vs. seladelpar 10 mg/daily vs. placebo for 52 weeks	Pruritus changed significantly from the baseline in seladelpar 10 mg arms vs. placebo (CI 95% −2.87–0.3, *p* = 0.02); however, pruritus was the most common AE in this arm (11%) similarly to the placebo arms (12.6%)	Upper abdominal pain, constipation, nausea, upper respiratory tract infections, headache, pruritus	Seladelpar was effective against pruritus. The study was prematurely terminated due to unexpected histological findings (i.e., portal inflammation and interface hepatitis with plasma cells, bile duct injury/cholangitis, vascular changes, and other miscellaneous findings) observed in a concurrent study of seladelpar in patients with NASH (NCT03551522)
	Hirschfield MG et al. [133] NCT04620733	ST: double-blind, placebo-controlled, multicenter RCT P: 193 patients with PBC with inadequate response to UDCA D: seladelpar 5 mg/daily vs. seladelpar 10 mg/daily vs. placebo/daily for 52 weeks	Among patients who exhibited moderate to severe pruritus, a statistically significant reduction in pruritus was observed between the baseline and after 6 and 12 months of treatment with seladelpar compared to a placebo	Headache, abdominal pain, nausea, abdominal distention	The statistically significant difference in pruritus reduction was observed only in patients with moderate to severe pruritus. Pruritus was assessed using different tools
**ELAFIBRANOR**	A Study of Elafibranor in Adults With Primary Biliary Cholangitis and Inadequate Response or Intolerance to Ursodeoxycholic Acid. (ELSPIRE) NCT06383403	ST: placebo-controlled, multicenter, double-blind RCT P: 72 patients with PBC D: elafibranor 80 mg/daily vs. placebo for 52 weeks, then follow-up for 4 weeks	Active, not yet recruiting	No results posted	Itching was assessed using the PBC Worst Itch Numeric Rating Scale (NRS), 5D itch score, and PBC-40 Quality of Life scale. Small population
	Kowdley K.V. et al. [129] (ELATIVE) NCT04526665	ST: placebo-controlled, multicenter, double-blind RCT P: 161 patients with PBC with inadequate response or intolerance to ursodeoxycholic acid D: elafibranor 80 mg/daily vs. placebo for 52 weeks-104 weeks, then elafibranor 80 mg/daily for 4–5 yrs	A statistically significant reduction in pruritus was observed in patients treated with elafibranor, as assessed with the pruritus domain of the PBC-40 and the 5D itch scale. However, no statistical difference was noted between the two groups when using the worst itch NRS	Abdominal pain, diarrhea, nausea, vomiting, and elevated creatine phosphokinase levels were observed	Itching was assessed using the PBC Worst Itch NRS Score ≥ 4 and PBC-40 scales. Reduction in itching is considered a secondary outcome. Conflicting results have been demonstrated regarding the effectiveness of treatments in reducing pruritus
	A Study Observing Everyday Effectiveness and Safety of the Drug Elafibranor in Participants With Primary Biliary Cholangitis Who Are Receiving Ongoing Treatment (ELFINITY) NCT06447168	ST: observational, prospective, non-interventional, muticenter P: 424 patients with PBC D: elafibranor 80 mg/day for 24 months	Active, not yet recruiting	No results posted	Itching was assessed using the PBC itch score, 5D itch scale, and PBC-40 scales
	Schattenberg JM et al. (GENFIT) NCT03124108	St: placebo-controlled, multicenter, double-blind RCT P: 45 patients with PBC and inadequate response to UDCA D: elafibranor 80 mg vs. elafibranor 120 mg vs. placebo, twice a day for 12 weeks	In the subgroup of patients experiencing itching, a reduction in itching was demonstrated in both groups receiving elafibranor compared to the baseline	No serious adverse events occurred in the placebo and elafibranor 80 mg groups. In the elafibranor 120 mg group, two patients experienced severe drug-related adverse events (stroke and autoimmune hepatitis)	Pruritus variations is a secondary outcome. Limited population size

Fibrates in cholestatic pruritus. UDCA = ursodeoxycholic acid, OCA = obeticholic acid.

### 5.2. K Opioid Receptor (KOR) Agonists

Nalfurafine, which has already been discussed earlier due to its approval in Japanese guidelines, belongs to the category of K opioid receptor agonists, as do difelikefalin and nalbuphine. Difelikefalin (DFK) is a KOR agonist with limited penetration into the central nervous system (CNS) that inhibits afferent transmission of sensory signals to the central nervous system [134]. It also appears to have immunomodulatory activity capable of reducing the production of proinflammatory cytokines [135]. The drug has recently been approved by the Food and Drug Administration (FDA) and the European Medicines Agency (EMA) as a first-line treatment for uremic pruritus in adults undergoing hemodialysis [136]. As for cholestatic pruritus, a study evaluating the efficacy and safety of the drug in patients with PBC and moderate to severe pruritus has been completed, although it experienced a slowdown in recruitment due to the COVID-19 pandemic [NCT03995212].

Nalbuphine is a semi-synthetic KOR agonist and partial mu-opioid receptor antagonist that has been approved by the FDA for the treatment of moderate-to-severe pain in patients requiring opioid therapy and in whom other treatments have failed. Currently, a phase 1 study is underway in patients with liver disease and pruritus [NCT04020016]. Table 7 reports the main clinical studies on KOR agonists in cholestatic itch.

### 5.3. Cannabinoids

Regarding the use of cannabinoids in cholestatic pruritus, two cases have been reported by Neff et al. of patients with refractory pruritus even after plasma exchange, including a woman with medroxyprogesterone-induced cholestatic disease and a 57-year-old woman with PBC treated with dronabinol, a synthetic analogue of tetrahydrocannabinol, the active ingredient in marijuana. Among the reported side effects was coordination disturbance, which was resolved by reducing the Marinol dosage to 2.5 mg/daily [137]. Table 8 reports the main clinical studies on cannabinoids in cholestatic itch.

### 5.4. Ileal Bile Acid Transport (IBAT) Inhibitors

A critical step in the enterohepatic circulation of bile acids is the almost complete reabsorption of conjugated bile acids in the ileum through the action of the apical sodium-dependent bile acid transporter (or ileal bile acid transporter) [138]. In the ileum, 95% of bile acids are reabsorbed by the IBAT and reintroduced into the portal circulation, where the sodium-dependent taurocholate cotransporting peptide (NTCP) brings them back into the liver [139]. IBAT inhibitors inhibit bile acid reabsorption at the ileal level. Additionally, the reduction in the enterohepatic circulation of bile acids leads to an increase in bile acid synthesis as a consequence of feedback on the intestinal FXR and subsequently the utilization of cholesterol for de novo bile acid synthesis, thereby reducing circulating LDL levels [138]. The presence of bile acids in the intestinal lumen increases intestinal peristaltic activity and diarrhea. For these reasons, IBAT inhibitors have been used in various diseases, including NASH, cholestatic pruritus, and the variant with predominantly constipation of irritable bowel syndrome. Regarding cholestatic pruritus, trials that have been conducted evaluated the effectiveness of maralixibat, odevixibat, and linerixibat.

Maralixibat has been primarily studied in progressive familial intrahepatic cholestasis (PFIC) and Alagille syndrome (ALGS). In the trials, pruritus was generally evaluated using the Itch Reported Outcome (ItchRO) instrument, a scale that assesses pruritus on a 5-point scale (0 = no pruritus, 4 = very severe pruritus).

Two additional RCTs, IMAGO and ITCH, and their extensions IMAGINE and IMAGINE II, respectively, evaluated the efficacy, tolerability, and reduction in pruritus with maralixibat in patients with Alagille syndrome, evidence of cholestasis, and moderate to severe pruritus [140]. In the phase 2 IMAGO trial (NCT01903460), no significant differences were observed between maralixibat and a placebo in changing pruritus scores based on the ItchRO scale [141], while the extensions IMAGINE, ITCH, and IMAGINE II demonstrated a reduction in pruritus in patients who took maralixibat compared to the baseline (NCT02057692, NCT02117713, and NCT02047318). Maralixibat has been approved by the FDA for cholestatic pruritus in patients with ALGS one year of age and older and by the EMA for cholestatic pruritus in patients with ALGS two months of age and older [142,143]. Recently, Hansen et al. compared the outcomes of patients with Alagille syndrome (ALGS) undergoing treatment with maralixibat for six years to those of patients in the Global Alagille Alliance (GALA) study as the open-label studies did not include a control group [144]. The study compared 84 ALGS patients treated with maralixibat to 469 individuals from the GALA study and assessed differences in the incidence of events such as transplantation, surgical biliary diversion, portal hypertension manifestations, and mortality. ALGS patients receiving maralixibat demonstrated increased event-free survival compared to the control patients from the GALA study (71.4% vs. 50.0%; *p* < 0.0001) and a 67% improvement in transplant-free survival [144].

The ICONIC trial and the INDIGO trial demonstrated a reduction in pruritus in patients with Alagille syndrome and PFIC, respectively [145,146]. Studies on progressive familial intrahepatic cholestasis (PFIC) have also shown promising results of maralixibat in reducing cholestatic pruritus, and further studies are ongoing. The table below summarizes the studies in which pruritus is evaluated as an outcome.

Odevixibat (A4250) is an inhibitor of the ileal bile acid transporter (IBAT) that has shown promising results in reducing pruritus. It has recently been approved by the FDA and EMA for the treatment of pruritus in patients 3 months of age and older with progressive familial intrahepatic cholestasis [147,148]. In the PEDFIC 1 RCT, a statistically significant reduction in pruritus was demonstrated in pediatric patients with PFIC 1 and 2 at different dosages [149]. An optional 72-week open-label extension study is still ongoing [PEDFIC 2, NCT03659916]. ALGS trials such as ASSERT-EXT [NCT05035030] are currently ongoing, while results from the ASSERT trial demonstrated a significant reduction in pruritus among ALGS patients treated with odevixibat compared to the placebo group after 24 weeks of therapy [150]. A trial on nine patients evaluated the efficacy and tolerance of odevixibat in patients with PBC and overlap PBC/autoimmune hepatitis [NCT02360852]; during the 4 weeks, adverse events likely related to the high drug dosage, such as abdominal pain and diarrhea, led to a high number of dropouts from the study [151].

Linerixibat has been tested for cholestatic pruritus in participants with PBC [152]. The results of this study showed a failure to achieve the primary endpoint, which was a statistically significant reduction in pruritus by linerixibat compared to a placebo. A phase 3 study evaluating the efficacy and safety of linerixibat in patients with PBC and cholestatic pruritus is currently recruiting patients [NCT04950127], and a compassionate use study of cholestatic pruritus in patients with PBC is also ongoing [NCT05448170]. Table 9 reports the main clinical studies on IBAT inhibitors in cholestatic itch.

## 6. Modulation of Other Itch Pathways

### 6.1. UDCA

UDCA is a bile acid that acts at the hepatic level in several ways, including altering the composition of bile acids, cytoprotection, and immunomodulation, although the exact mechanism is not well understood [155]. UDCA is able to significantly reduce the concentration of cholesterol in bile by inhibiting its intestinal absorption and secretion into the bile [156]. Cytoprotection at the level of hepatocytes and cholangiocytes occurs through protection against bile acids, which are capable of generating reactive oxygen species that can cause an inflammatory response [157]. Additionally, UDCA is able to increase bile acid secretion [158].

UDCA is considered the first-line drug in PBC and PSC, and it is also prescribed in ICP. However, for these two conditions, there are no studies that have focused on pruritus as a primary endpoint. Several trials have evaluated the effectiveness of UDCA in reducing pruritus in ICP [159,160,161]. The PITCHES trial is a double-blind, multicenter, randomized, placebo-controlled trial that enrolled 605 women, half of whom took UDCA and half took a placebo, and evaluated the potential reduction in perinatal outcomes induced by UDCA [162]. No significant differences in perinatal outcomes were observed between the two groups; however, a statistically significant reduction in the perception of pruritus was observed in the arm that received UDCA [162]. Meta-analyses have also shown that UDCA reduces pruritus during ICP, and doses up to 20 mg/kg/day do not have a toxic effect on the mother or fetus [163]. UDCA is recommended as first-line therapy in PSC and PBC for its anticholestatic effect, but no effects on pruritus have been reported, and therefore it is not recommended by guidelines as a treatment for pruritus [3,6,8].

Tauroursodeoxycholic acid (TUDCA), a more hydrophilic metabolite of UDCA, was used in a study involving 40 patients with cirrhosis, primarily of viral etiology, resulting in a significant reduction in pruritus in the experimental group compared to a placebo (*p* < 0.05) [164]. A recent analysis of early signs in PBC highlighted the possibility that elevated concentrations of lipophilic bile acids may damage the cell membranes of various cell types [165]. It is possible to hypothesize, based on these data, that the use of more hydrophilic bile acids, such as TUDCA, may reduce cholestatic pruritus. Further studies in this direction are warranted. Table 10 reports the main clinical studies on UDCA in cholestatic itch.

### 6.2. Antihistamine

The role of histamine in pruritus has been well documented, and therefore its involvement in cholestatic pruritus cannot be completely ruled out. However, it has been noted that high concentrations of bile acids, especially lipophilic ones, are required to induce histamine release from mast cells [166] and that antihistamines have shown little efficacy in the treatment of cholestatic pruritus [102,167,168].

### 6.3. S-Adenosyl Methionine (SAMe)

SAMe is the primary biological methyl donor synthesized by mammalian cells, with 85% of SAMe being synthesized by the liver [169]. SAMe plays an important cytoprotective role against oxidative stress. SAMe may also contribute to the formation of sulfated bile acids, which could be excreted more efficiently by the kidneys compared to their non-sulfated counterparts [170]. Furthermore, it appears that a greater improvement in pruritus may be achieved when SAMe is administered parenterally [170]. A study conducted on 24 PBC patients treated with UDCA and 1200 mg/daily of SAMe for 6 months demonstrated a statistically significant reduction in pruritus compared to the baseline [171]. Further studies are needed to confirm the efficacy of SAMe in the treatment of pruritus associated with PBC and to understand the underlying mechanism of this therapeutic effect. Table 11 reports the main clinical study on UDCA in cholestatic itch.

### 6.4. Dupilumab

Dupilumab is a monoclonal antibody of immunoglobulin subclass G4 directed against the alpha receptor of interleukin 4 (IL-4), thus acting as its antagonist [172]. The alpha receptor of IL-4 is also shared by IL-13, so dupilumab inhibits the signaling of both IL-4 and IL-13, inducing the release of proinflammatory cytokines, chemokines, and immunoglobulin E [172]. Currently, the drug is approved for moderate to severe atopic dermatitis, severe asthma, chronic sinusitis with nasal polyps, prurigo nodularis, and eosinophilic esophagitis [173]. A phase 2, open-label study lasting 18 weeks in patients with moderate to severe hepatic-origin pruritus, which involves administering 300 mg of dupilumab subcutaneously every 2 weeks, is currently ongoing [NCT04256759].

## 7. Conclusions

For about 2000 years, it was believed that the origin of pruritus in jaundiced patients could be bile particles [174], and this paradigm has been increasingly enriched by new insights and findings. It has been shown that in vivo levels of bile acids do not correlate with pruritus and that they are not able to activate some membrane or nuclear receptors involved in the pruritus pathway. Furthermore, cholestatic pruritus differs markedly from classical pruritus due to its poor response to antihistamine therapy. Cholestatic pruritus negatively affects the quality of life of patients suffering from it, and finding a solution that can reduce or resolve this symptom is imperative for the physician. Despite the immense progress made by research in unraveling the mechanisms of pruritus, only the surface has been scratched at present. Animal models and initial human studies have highlighted a complex world of interconnections and unexpected correlations between pruritogens, receptors, and physiological conditions. The proposed guideline treatments are many, and therefore, it is necessary to proceed step by step in identifying the best treatment for each patient. Although the latest EASL and AASLD guidelines have proposed a sequence for selecting different molecules for the treatment of cholestatic pruritus, the scientific evidence supporting most of the mentioned drugs is limited and based on case reports or studies with small, non-randomized populations. Additionally, some of the drugs used may have significant side effects or interactions with other molecules. Therefore, the treatment of cholestatic pruritus should involve a personalized approach, often requiring sequential use of medications until an effective drug for pruritus relief is found. However, in some cases, pruritus may be refractory to any recommended therapy. Studies involving IBAT and PPAR give hope for reducing pruritus in cholestatic diseases, although they are burdened with frequent gastrointestinal side effects. These drugs have not shown interaction with UDCA, the drug of choice in many cholestatic diseases, and can be used in synergy with it. As for PPAR, studies are underway on larger cohorts.

Among the most frequently encountered limitations in studies on these drugs are the small size of the study populations and the lack of a placebo control or randomization. Further studies in wider populations are needed to assess the real efficacy and tolerability in long-term drug use.

Studies on the role of autotaxin and TRPV will be essential in better characterizing the genesis of cholestatic pruritus and in the development of drugs for this symptom in the coming years.

## Figures and Tables

**Table 1:** Bile sequestrants used or tested for cholestatic itch.

Drug	Dosage	Comment	Side Effects	Ref.
**Cholestyramine**	4–16 g/daily	There is no significant scientific evidence of its use	Unpleasant taste, abdominal discomfort, constipation, bloating, alteration in absorption of other drugs and vitamins	[64,65]
**Colestipol**	4–16 g/daily	Off-label use; no scientific evidence supporting the use	Unpleasant taste, abdominal discomfort, constipation, bloating, alteration in absorption of other drugs and vitamins	
**Colesevelam**	1875 mg twice daily	Off-label use; ineffective in relief from pruritus	Constipation, reduction in bioavailability of some drugs	[66]

**Table 2:** Rifampicin in cholestatic pruritus. INR= international normalized ratio.

Drug	Dosage	Comment	Side Effects	Ref.
**Rifampicin**	150–600 mg/daily	Strongly recommended, different RCT show efficacy in pruritus reduction	Nausea, anorexia, hepatitis, change in urine color, intestinal microbiome alteration, elevation of bilirubin, prolongation of INR, induction of cytochrome P450	[69,70,71,72]

**Table 3:** Most common opioid antagonists used for cholestatic itch.

Drug	Dosage	Comment	Side Effects	Ref.
**Naltrexone**	12.5–25 mg/daily until 50 mg/daily PO	Off-label use; RCTs showed a reduction in itch in patients with other therapy failure	Withdrawal syndrome, headache, vomiting, dizziness, uncontrolled pain in patients with underlying chronic pain, rare cases of hepatitis	[78,79]
**Naloxone**	From 0.002 µg/kg/min (starting dose) to 0.2 µg/kg/min IV	Off-label use; there are only endovenous formulations, and thus it can be used only in hospital settings	Withdrawal syndrome, headache, vomiting, dizziness, uncontrolled pain in patients with underlying chronic pain. Breakthrough phenomenon	[50,80]
**Nalmefene**	5 mg BID with gradual increase until 40 mg TID PO	Off-label use; good tolerance and efficacy	Withdrawal syndrome, headache, vomiting, dizziness, uncontrolled pain in patients with underlying chronic pain	[51]
**Nalfurafine**	2.5–5 µg/daily PO	Licensed for pruritus only in Japan, good tolerance and good efficacy	Pollakiuria (including nocturia), somnolence, insomnia (including middle insomnia), and constipation	[81,82,83,84]
**Butirphanol**	1–2 mg/daily intranasal spray	Off-label use; only case report studies showing beneficial effects, little evidence	somnolence, sedation, nausea, vomiting, and dizziness	[85]

PO = per oral, IV = intravenous.

**Table 4:** Modulators of serotoninergic pathways tested for cholestatic pruritus.

Drug	Dosage	Comment	Side Effects	Ref.
**Sertraline**	25 mg/daily (starting dose) until 100 mg/daily	Off-label use; RCTs showed a reduction in itch. Not better efficacy then rifampicin but probably better safety profile.	dry mouth, insomnia, increased appetite, somnolence, headache, visual hallucinations	[75,88]
**Ondansetron**	8 mg intravenously	Off-label use; failure in ameliorating pruritus	Headache, flush, constipation,	[58,59,60]
**Phenobarbital and Propofol**	For propofol, 15 mg intravenously	Off-label use; not, no safety and efficacy profiles available	Excessive sedation, respiratory failure, hepatotoxicity	[9,69,90,91]
**Gabapentin**	300–1800 mg/daily	Off-label use: failure in ameliorating pruritus liver related, further studies are needed	Respiratory infection, viral infection, depression, anxiety, somnolence, ataxia, dizziness	[92]

**Table 5:** Physical approaches for cholestatic itch.

Procedure	No. of Average Treatments	Comment	Side Effects	Ref.
**MARS**	Two procedures 2 days apart	Off-label use, small studies, non-randomized and placebo-controlled trial. Good efficacy and tolerability. High cost, available in few centers, requires trained staff	Temporary reduction in platelet count and hemoglobin level	[95,96,98,99,100]
**UV light therapy**	26 procedures	Off-label use; good tolerability and safety	Erythema and paresthesia	[103,104]
**Plasmapheresis**	1–6 hospital admissions in which 4–6 procedures were performed	Off-label use; good tolerance and efficacy. Great reduction in pruritus	Transient hypotension and urticaria	[105,106]
**Naso-biliary drainage**	Median duration of 7 days	Licensed for pruritus only in Japan, good tolerance and good efficacy	Pancreatitis and cholangitis	[107]
**Charcoal hemoperfusion**	5 sessions	Off-label use; effective in reduction in pruritus	Fever, pain, bleeding from the catheter site	[108]

**Table 7:** Main clinical studies on KOR agonists in cholestatic itch.

Drug	Reference	Study Type (ST), Population (P), and Dosage (D)	Results	Adverse Effects (AEs)	Limitations/Comments
**DIFELIKEFALIN**	Study to Evaluate the Safety and Efficacy of Oral CR845 (Difelikefalin) in Patients with Primary Biliary Cholangitis (PBC) and Moderate-to-Severe Pruritus NCT03995212	ST: double-blind, placebo-controlled RCT, multicenter P: 14 patients with PBC with moderate-to-severe pruritus D: difelikefalin 1.0 mg twice/daily vs. placebo for 16 weeks	No results posted	No results posted	Terminated, slow enrolment due primarily to COVID-19, phase 2 study, small populations
**NALBUPHINE**	Nalbuphine ER Effects of Liver Disease on Pharmacokinetics and Itch NCT04020016	ST: interventional, non-randomized P: 56 patients with liver cirrhosis and healthy subjects D: two arms: in the first, single ascending doses are administered and observed for 4 days; in the second, multiple ascending doses are administered until the 13th day	Active, not recruiting, no results posted	No results posted	Not well explicated which liver diseases were considered. Pruritus is a secondary outcome

**Table 8:** Main clinical studies on cannabinoids in cholestatic itch.

Drug	Reference	Study Type (ST), Population (P), and Dosage (D)	Results	Adverse Effects (AEs)	Limitations/Comments
**CANNABINOIDS**	Efficacy and Tolerance of Cannabidiol in Patients With Severe Pruritus: a Multicenter, Double-blind, Randomized, Placebo- controlled Study (CANNABITCH) NCT06435299	ST: double-blind, placebo controlled, phase 3 RCT P: estimated 218 patients with severe pruritus, regardless of the cause of the pruritus D: cannabis oil 50 mg/mL arm: an auto-titration phase during the first 14 days of treatment: 0.2 mL on the first day then an increase of 0.2 mL every 2 days in two daily doses, with 1.4 mL/day maximum vs. placebo arm	No results posted	No results posted	Severe pruritus, regardless of the cause of the pruritus

**Table 9:** Main clinical studies on IBAT inhibitors in cholestatic itch.

Drug	Reference	Study Type (ST), Population (P), and Dosage (D)	Results	Adverse Effects (AEs)	Limitations/Comments
**MARALIXIBAT**	Loomes KM et al. [146] (INDIGO) NCT02057718	ST: interventional, single group assignment, open-label P: 33 children with PFIC D: maralixibat 266 μg/kg/daily for 72 weeks then twice daily for 240 weeks	Among responders, all experienced a > 1.0-point clinically meaningful reduction in ItchRO(Obs) scores	Gastrointestinal disorders such as abdominal pain, diarrhea, and gastroenteritis (almost 80%)	Lack of a placebo-controlled element and the relatively small sample size
	Gonzales E et al. [145] (ICONIC) NCT02160782	ST: placebo-controlled, multicenter, double-blind RCT with randomized withdrawal period (RWD), phase 2b study with a long-term, open−label extension P: 31 children with clinical diagnosis of Alagille syndrome D: six phases: 6 weeks of dose escalation then 12 weeks of stable dose (up to 380 μg/kg/daily), then 4 randomized weeks vs. placebo, then maralixibat 380 μg/kg/daily for 26 weeks and after week 100 double daily maralixibat doses in low-responder patients	Significant improvements in different pruritus scales (ItchRO[Obs], ItchRO[Pt], and CSS scales). An increase in pruritus was observed in the placebo group with a subsequent decrease with the resumption of therapy	Most adverse events were self-limiting and mild to moderate. Diarrhea and abdominal pain were the most frequent. A total of 29% of participants experienced deficiency of Vitamin D	Duration of placebo-controlled trial was only 4 weeks, small population. Long duration of study
	Shneider BL, et al. [141] (ITCH) NCT02057692	ST: double-blind, placebo-controlled RCT P: 37 children with clinical diagnosis of Alagille syndrome, evidence of cholestasis and moderate to severe pruritus D: four arms: 70 µg/kg/daily vs. 140 µg/kg/daily vs. 280 µg/kg/daily vs. placebo for 13 weeks	Reduction in ItchRO(Obs) was not significant between maralixibat groups and placebo. Reductions in reported pruritus were observed in the 70 and 140 µg/kg/daily groups. A reduction in clinician reported pruritus was more common in maralixibat groups vs. placebo groups	AEs were similar in the maralixibat and in placebo groups	Small study population and short duration of the study
	An Extension Study to Evaluate the Long-Term Safety and Durability of Effect of LUM001 in the Treatment of Cholestatic Liver Disease in Pediatric Subjects With Alagille Syndrome (IMAGINE-II) NCT02117713	ST: interventional, single group assignment, multicenter P: 34 children with clinical diagnosis of Alagille syndrome, evidence of cholestasis and moderate to severe pruritus which had completed ITCH trials D: 280 mcg/kg/daily for 220 weeks	A significant reduction in pruritus was reported	Abdominal discomfort, lymphadenopathy, Diarrhea, gastroesophageal reflux disease	Difference was only calculated in participants who had baseline and week 218 values for six participants
	Safety and Efficacy Study of LUM001 in the Treatment of Cholestatic Liver Disease in Patients With Alagille Syndrome (IMAGO) NCT01903460	ST: double-blind, placebo-controlled RCT, P: 20 children with clinical diagnosis of Alagille syndrome, evidence of cholestasis and moderate to severe pruritus D: four arms: 140 µg/kg/daily vs. placebo vs. 280 µg/kg/daily vs. placebo for 13 weeks	No significant reduction in ItchRO between maralixibat and the placebo was reported	Abdominal pain, diarrhea, nausea	Small study population
	An Extension Study to Evaluate the Long-Term Safety and Durability of Effect of LUM001 in the Treatment of Cholestatic Liver Disease in Subjects With Alagille Syndrome (ALGS) (IMAGINE) NCT02047318	ST: interventional, open-label, single group assignment P: 19 children with clinical diagnosis of Alagille syndrome, evidence of cholestasis and moderate to severe pruritus which completed IMAGO trial D: up to 560 µg/kg/daily for 288 weeks	Significant reduction and durability of pruritus assessed using ItchRO scale from baseline	Gastrointestinal disorders as abdominal pain (47%), diarrhea (1.6%), abnormal feces, flatulence, nausea (5.3%)	Not placebo-controlled, small population
	MRX-800: A Long-Term Safety Study of Maralixibat in the Treatment of Cholestatic Liver Disease in Subjects Who Previously Participated in a Maralixibat Study (MERGE) NCT04168385	ST: interventional, open-label, multicenter P: 52 participants previously participated in a maralixibat study D: maralixibat up to 1200 µg/kg/daily for 3 years	Active, not recruiting, no result posted	No result posted	Not placebo-controlled; pruritus evaluation is a secondary outcome
	Miethke et al. [153] NCT03905330	ST: placebo-controlled, multicenter RCT P: 93 participants aged 1–17 years with PFIC and persistent pruritus D: maralixibat up to 570 µg/kg/daily for 26 weeks vs. placebo	Significant reduction in ItchRO(Obs) in patients with bile salt export pump deficiency in FIC1, MDR3, TJP2, MYO5B, and in entire PFIC cohort	Diarrhea (57.4%) was the most common AE in maralixibat group. Severe AEs were similar between the two groups	Small study population
	A Study of TAK-625 for the Treatment of Progressive Familial Intrahepatic Cholestasis (PFIC) NCT05543187	ST: interventional, open-label, single group assignment P: nine Japanese children aged more than 1 month with PFIC, cholestasis, and pruritus D: Increasing dose of maralixibat up to 600 µg/kg/twice daily for 34 months	Recruiting	No results posted	Small study population, no RCT, change in the Average Morning ItchRO (Obs) Severity Score is a primary outcome
	An Open-label Extension Study to Evaluate the Long-term Safety and Efficacy of Maralixibat in the Treatment of Subjects With Progressive Familial Intrahepatic Cholestasis (PFIC) NCT04185363	ST: interventional, open-label, single group assignment, multicenter, interventional, phase 3 P: 90 patients who have completed study MRX-502 D: 600 mcg/kg twice daily	Active, not recruiting	No results posted	Small study population, no RCT
	A Study of TAK-625 for the Treatment of Alagille Syndrome (ALGS) NCT05543174	ST: interventional, open-label, single group assignment P: five Japanese children aged more than 1 month with ALGS, cholestasis, and pruritus D: starting dose of maralixibat of 200 µg/kg/daily for 1 week then 400 µg/kg/daily for 34 months	Recruiting	No results posted	Change in weekly average severity of pruritus measured with ItchRO (Obs) from the baseline to week 18 is a secondary outcome
**ODEVIXIBAT**	Ovchinsky N. et al. [150] NCT04674761	ST: placebo-controlled, multicenter RCT P: 52 patients with genetically confirmed ALGS, cholestasis, and significant pruritus D: odevixibat 120 µg/kg/day vs. placebo for 24 weeks	A statistically significant reduction was observed in the group taking odevixibat compared to the placebo after 24 weeks (*p* = 0.0024). Pruritus was assessed with ObsRO scratching score	The most common adverse AE in interventional group was diarrhea (29%)	Small study population
	Long-term Safety and Efficacy of Odevixibat in Patients with Alagille Syndrome (ASSERT-EXT) NCT05035030	ST: interventional, open-label, single group assignment P: 63 patients that terminated the 24-week treatment period of study A4250-012 [ASSERT] D: treatment of 72 weeks (cohort 1) or 12 weeks (cohort 2). Odevixibat 120 μg/kg/day	Active, not recruiting	No result posted	Change from the baseline in scratching score as measured using the Albireo Observer-Reported Outcome Caregiver Instrument is the primary outcome. Not an RCT, small population
	Al-Dury S, et al. [151] NCT02360852	ST: interventional, open-label, single group assignment P: nine adult patients with PBC or PBC-autoimmune hepatitis overlap, UDCA non-responders, with moderate pruritus treated with anion exchange resins D: odevixibat 0.75–1.5 mg/daily for 4 weeks	Terminated with high side effects incidence (diarrhea, abdominal pain). Pruritus improvement was reported	Abdominal pain, diarrhea, melena and significantly decreased hemoglobin (one patients)	Small study population, probably excessive dose
	Thompson RJ et al., [149] (PEDFIC 1) NCT03566238	ST: randomized, double-blind, placebo-controlled, multicenter P: 62 children with clinical and genetically confirmed PFIC 1 or 2, cholestatic and significant pruritus D: three arms: odevixibat 40 μg/kg/daily vs. 120 μg/kg/daily vs. placebo for 24 weeks	Intervention showed statistically significant improvements in pruritus compared with the placebo based on ObsRO instrument measurements	Diarrhea (31% vs. 10%), vomiting (17% vs. 0%), abdominal pain (7% vs. 0%), ALT increase (10 vs. 5%)	Small population
	Long Term Safety & Efficacy Study Evaluating The Effect of A4250 in Children With PFIC (PEDFIC 2) NCT03659916	ST: interventional, open-label, single group assignment P: 116 children with clinical and genetically confirmed PFIC 1 or 2, cholestatic and significant pruritus and patients that completed or withdrawn PEDFIC 1 D: odevixibat 40 μg/kg/daily or 120 µg/kg/daily for 72 weeks, or 40 µg/kg/day for the first 12 weeks followed by 120 µg/kg/day for the remaining 60 weeks	Active, not recruiting	No result posted	Small population, not an RCT, change in pruritus as indexed by caregiver report (Albireo ObsRO instrument). Observed scratching is a primary endpoint
	Odevixibat for the Treatment of Progressive Familial Intrahepatic Cholestasis NCT04483531	ST: expanded-access program for patients with PFIC in the US who have pruritus and elevated serum bile acids and who are not able to be enrolled in A4250-008 (PEDFIC2) P: nine children with PFIC, cholestatic, and significant pruritus D: odevixibat 120 μg/kg/day	Expanded-access program, approved for marketing	No results posted	Expanded-access program
	Baumann et al. [154] NCT02630875	ST: interventional, open-label, single group assignment P: 22 children with diagnosis of pruritus to chronic cholestasis but also with PFIC, ALGS, biliary atresia, and sclerosing cholangitis D: different arms with six different odevixibat doses for 4 weeks	An improvement in mean itching was observed in all study arms, with reductions noted across various disease subgroups	Ear infection (12.5%) and pyrexia (12.5%),	Small population, No RCT, not placebo-controlled. Pruritus variations are a secondary outcome
**LINERIXIBAT**	Levy C, et al. [152] GLIMMERS NCT02966834	ST: placebo-controlled, multicenter, double-blind RCT P: 147 patients with PBC and moderate to severe pruritus D: linerixibat 20 mg/daily vs. linerixibat 90 mg/daily vs. linerixibat 180 mg/daily vs. linerixibat 40 mg/twice daily vs. linerixibat 90 mg/twice daily vs. placebo for 16 weeks	A significant change from the baseline in monthly itch score was noted among linerixibat 180 mg/daily, 40 mg/twice daily and 90 mg/twice daily vs. placebo. A significant relationship between total daily dose and response was observed post hoc in the per protocol population	Diarrhea, abdominal pain	Linerixibat’s effect on itch was not significantly different versus the placebo in the primary intent-to-treat analysis. High placebo response
	Global Linerixibat Itch Study of Efficacy and Safety in Primary Biliary Cholangitis (PBC) (GLISTEN) NCT04950127	ST: placebo-controlled, multicenter, double-blind RCT P: 238 patients with PBC and moderate to severe pruritus D: linerixibat 40 mg vs. placebo vs. linerixibat 40 mg followed by placebo vs. placebo followed by linerixibat 40 mg	Still recruiting, no results posted	No results posted	The effective drug dosage was not available
	Long-term Safety and Tolerability Study of Linerixibat for the Treatment of Cholestatic Pruritus in Participants With Primary Biliary Cholangitis (PBC) (LLSAT) NCT04167358	ST: open-label, non-comparator, global, multicenter, long-term safety study P: Participants who previously participated in the phase 2 studies (BAT117213 and 201,000 GLIMMER [group 1]) and phase 3 study (212620 GLISTEN [group 2]). D: linerixibat 40 mg twice/day	Active, still recruiting, no results posted	No results posted	Among the secondary outcomes is the assessment of itching using the Monthly Itch Scale
	Linerixibat Compassionate Use for Cholestatic Pruritus Adult Patients with Primary Biliary Cholangitis (PBC) NCT05448170	ST: expanded access, compassionate use patients with cholestatic pruritus due to PBC P: patients with cholestatic pruritus due to PBC D: linerixibat 40 mg	Active, compassionate use	No results posted	Compassionate use program

BSEP = bile salt export pump, FIC1 = familial intrahepatic cholestasis-associated protein 1, MDR3 = multidrug resistant 3 protein, TJP2 = tight junction protein 2, MYO5B myosin VB.

**Table 10:** UDCA for cholestatic itch.

Drug	Dosage	Comment	Side Effects	Ref.
**UDCA**	13–15 until 20 mg/Kg/daily	Off-label use for cholestatic itch: improvement of pruritus in ICP, no teratogenic activity	Pasty stools to diarrhea	[159,160,161]

**Table 11:** Main clinical studies on SAMe in cholestatic itch.

Drug	Reference	Study Type (ST), Population (P), and Dosage (D)	Results	Adverse Effects (AEs)	Limitations/Comments
**SAMe**	Wunsh E et al. [171] NCT02557360	ST: interventional, monocenter, open-label, single group assignment P: 24 patients with PBC D: SAMe 1200 mg/daily	Significant reduction in pruritus from baseline	No adverse events reported	Small study, no RCT, no placebo, pruritus was measured with PBC-40 questionnaire

## Data Availability

No new data were created.
